# Modeling non‐linear kinetics of hyperpolarized [1‐^13^C] pyruvate in the crystalloid‐perfused rat heart

**DOI:** 10.1002/nbm.3464

**Published:** 2016-01-18

**Authors:** E. Mariotti, M. R. Orton, O. Eerbeek, J. F. Ashruf, C. J. Zuurbier, R. Southworth, T. R. Eykyn

**Affiliations:** ^1^ Department of Imaging Chemistry and Biology, Division of Imaging Sciences and Biomedical EngineeringKing's College London, King's Health Partners *St. Thomas*' *Hospital* LondonUK; ^2^ CR‐UK and EPSRC Cancer Imaging Centre, Division of Radiotherapy and ImagingThe Institute of Cancer Research and Royal Marsden NHS TrustSuttonSurreySM2 5NGUK; ^3^ Department of Anatomy, Embryology and PhysiologyAMC, UvAAmsterdamThe Netherlands; ^4^ Laboratory Experimental Intensive Care Anesthesiology (LEICA), Department AnesthesiologyAMC, UvAAmsterdamThe Netherlands; ^5^ The British Heart Foundation Centre of Research ExcellenceThe Rayne Institute, King's College London, St. Thomas' HospitalLondonUK

**Keywords:** hyperpolarized ^13^C, dynamic nuclear polarization (DNP), cardiac metabolism, Langendorff perfused heart

## Abstract

Hyperpolarized ^13^C MR measurements have the potential to display non‐linear kinetics. We have developed an approach to describe possible non‐first‐order kinetics of hyperpolarized [1‐^13^C] pyruvate employing a system of differential equations that agrees with the principle of conservation of mass of the hyperpolarized signal. Simultaneous fitting to a second‐order model for conversion of [1‐^13^C] pyruvate to bicarbonate, lactate and alanine was well described in the isolated rat heart perfused with Krebs buffer containing glucose as sole energy substrate, or glucose supplemented with pyruvate. Second‐order modeling yielded significantly improved fits of pyruvate–bicarbonate kinetics compared with the more traditionally used first‐order model and suggested time‐dependent decreases in pyruvate–bicarbonate flux. Second‐order modeling gave time‐dependent changes in forward and reverse reaction kinetics of pyruvate–lactate exchange and pyruvate–alanine exchange in both groups of hearts during the infusion of pyruvate; however, the fits were not significantly improved with respect to a traditional first‐order model. The mechanism giving rise to second‐order pyruvate dehydrogenase (PDH) kinetics was explored experimentally using surface fluorescence measurements of nicotinamide adenine dinucleotide reduced form (NADH) performed under the same conditions, demonstrating a significant increase of NADH during pyruvate infusion. This suggests a simultaneous depletion of available mitochondrial NAD^+^ (the cofactor for PDH), consistent with the non‐linear nature of the kinetics. NADH levels returned to baseline following cessation of the pyruvate infusion, suggesting this to be a transient effect. © 2016 The Authors. *NMR in Biomedicine* published by John Wiley & Sons Ltd.

**Abbreviations used**
*α‐KG*: *α‐ketoglutarate*
*Ala*: *alanine*
*ALT*: *alanine transaminase*
*Asp*: *aspartate*
*Co*.: *cofactor*
*CoA*: *coenzyme A*
*DNP*: *dynamic nuclear polarization*
*EDTA*: *e* *thylenediaminetetraacetic acid*
*Glu*: *glutamate*
*HR*: *heart rate*
*KHB*: *Krebs*–*Henseleit buffer*
*Lac*: *lactate*
*LDH*: *lactate dehydrogenase*
*LVDP*: *left ventricular developed pressure*
*NAD*^+^: *nicotinamide adenine dinucleotide*
*NADH*: *nicotinamide adenine dinucleotide reduced form*
*PDH*: *pyruvate dehydrogenase*
*ppm*: *parts per* *million*
*Pyr*: *pyruvate*
*PyrH*: *pyruvate hydrate*
*SEM*: *standard error of the mean*
*T*_*R*_: *repetition time*.

## Introduction

The heart is a highly metabolically active and energy demanding organ that is able to utilize a variety of energy substrates, dependent upon their relative abundance, prevailing hormonal conditions, workload and oxygen availability 1. A number of experimental methods are available for investigating cardiac metabolism, including the use of radioactive tracer approaches 2, 3 and steady‐state incorporation of ^13^C labeled metabolites 4, 5. It has recently been shown to be possible to probe cardiac metabolism in real time (both *in vivo* and *ex. vivo* in Langendorff preparations), by injecting hyperpolarized [1‐^13^C] pyruvate and observing its conversion to bicarbonate, lactate and alanine by NMR or MRSI 6, 7, 8, 9. Fitting of the hyperpolarized ^13^C MRS time series of pyruvate and its metabolites to mathematical models 10, 11 gives estimates of rate constants for their interconversion. The rate constants are dependent on a range of factors, including substrate 12 and cofactor supply 13, membrane transport into or out of the cell 14, 15, enzyme expression levels and enzyme activities 16. A previous study has shown that under conditions approximating the fed state in the perfused heart infusion of 3 mM pyruvate contributed 80% of the resulting acetyl coenzyme A (CoA), while under fasting conditions (6) mM pyruvate contributed only 33% of the acetyl CoA, as measured with steady‐state incorporation of ^13^C labeled substrates, and concluded that studies of cardiac metabolism using hyperpolarized [1‐^13^C] pyruvate are highly sensitive to substrate competition 17.

The mathematical models employed to date typically use systems of modified Bloch equations 18 to fit the MR visible signals to first‐order kinetics. These models do not make allowances for any variation in rate constants *during* an experiment, such as substrate or product inhibition, changes in cofactor concentration, shifts in enzyme equilibria or perturbation of enzyme kinetics during substrate infusion. This is potentially problematic, because, despite the enhancements in sensitivity that this approach affords, it still requires infusion of hyperpolarized substrates at supra‐physiological concentrations. It has previously been shown that infusion of pyruvate in the perfused heart leads to an increase in nicotinamide adenine dinucleotide reduced form (NADH) 19, 20, and in the case of lactate dehydrogenase (LDH) it has been widely reported that this enzyme is susceptible to substrate inhibition at high pyruvate concentration 21, 22. The impact of the use of these non‐physiological concentrations upon the measured rate constants using first‐order models has previously been studied for hyperpolarized pyruvate *in vivo* in the heart, and showed a linear dependence against pyruvate dehydrogenase (PDH) activity measured by enzyme assay 10. However, the question of whether any transient time‐dependent effects are observed in the heart during substrate infusion has not been addressed.

In this work, we describe a non‐linear modeling approach to describe the kinetics of hyperpolarized [1‐^13^C] pyruvate conversion to lactate, bicarbonate and alanine in the perfused rat heart, which allows us to address this issue. The proposed model agrees with the principle of conservation of mass, where the hyperpolarized magnetization of the input function, representing the pyruvate bolus, and each metabolite decays to a non‐hyperpolarized or ‘MR invisible’ pool of the same metabolite 23. The total pool size of a given metabolite is therefore constant and proportional to concentration, enabling inclusion of apparent enzyme cofactor concentrations in the model. This formulation allows the modeling of possible non‐linear time‐dependent kinetics during the infusion of pyruvate, and can potentially be extended to other hyperpolarized substrates.

We studied two groups of hearts, one perfused with a standard Krebs–Henseleit buffer (KHB), where glucose was the only source of carbohydrate, and a second group perfused with KHB containing glucose supplemented with pyruvate. Non‐linear modeling suggests that infusion of the supra‐physiological concentrations of pyruvate necessary to perform the ^13^C dynamic nuclear polarization (DNP) NMR experiment perturbed the enzyme equilibria for LDH and alanine transaminase (ALT), and the steady‐state flux through PDH in the perfused heart, although in the case of LDH and ALT we were not able to distinguish between first‐ and second‐order models. In further experiments using fluorescence measurements from the surface of the heart, we demonstrate that these non‐linear variations in flux can be explained, in part, by exhaustion of mitochondrial nicotinamide adenine dinucleotide (NAD^+^) by the supra‐physiological concentrations of pyruvate employed.

## Materials and Methods

### Modeling higher‐order kinetics of hyperpolarized substrates

The simplest model of differential equations that describes first‐order one‐way kinetics of a simple reaction of hyperpolarized substrate A* → B* can be written.

(1) $${\mathrm{dA}}^{*}\left(t\right)/dt=-k_{\mathrm{AB}}A^{*}\left(t\right)-r_{1A}A^{*}\left(t\right)$$

(2) $${\mathrm{dB}}^{*}\left(t\right)/dt=k_{\mathrm{AB}}A^{*}\left(t\right)-r_{1B}B^{*}\left(t\right)$$

where * denotes hyperpolarized substrate pools, *k*_AB_ denotes the first‐order rate constant, *r*_1A_ and *r*_1B_ represent the effective longitudinal relaxation rates of A* and B* given by *r*_1A_ = 1/*T*_1A_ − *T*_R_^−1^ ln (cos*θ*) and *r*_1B_ = 1/*T*_1B_ − *T*_R_^−1^ ln (cos*θ*), taking into account the loss of signal due to *T*_1_ and the application of the RF excitation pulse characterized by a flip angle *θ* and repetition time *T*_R_ 24.

The model can be extended so that the hyperpolarized pool of substrates A* and B* relax to an ‘MR invisible’ non‐hyperpolarized pool of reactants A and B 23:

(3) $$\mathrm{dA}\left(t\right)/dt=-k_{\mathrm{AB}}A\left(t\right)+r_{1A}A^{*}\left(t\right)$$

(4) $$\mathrm{dB}\left(t\right)/dt=k_{\mathrm{AB}}A\left(t\right)+r_{1B}B^{*}\left(t\right).$$

The above system of Equations [(1)]–[(4)] agrees with the principle of conservation of mass, since the rate of change d[A* + A + B* + B]/d*t* is zero.

This system of differential equations can be readily extended to account for higher order reaction kinetics. For example, consider a second‐order reaction A* + Co. → B*, where Co. represents a common reaction cofactor that is non‐hyperpolarized, to give a system of differential equations:

(5) $${\mathrm{dA}}^{*}\left(t\right)/dt=-k_{\mathrm{AB}}A^{*}\left(t\right)\mathrm{Co}\left(t\right)-r_{1A}A^{*}\left(t\right)$$

(6) $${\mathrm{dB}}^{*}\left(t\right)/dt=k_{\mathrm{AB}}A^{*}\left(t\right)\mathrm{Co}\left(t\right)-r_{1B}B^{*}\left(t\right)$$

(7) $$\mathrm{dA}\left(t\right)/dt=-k_{\mathrm{AB}}A\left(t\right)\mathrm{Co}\left(t\right)+r_{1A}A^{*}\left(t\right)$$

(8) $$\mathrm{dB}\left(t\right)/dt=k_{\mathrm{AB}}A\left(t\right)\mathrm{Co}\left(t\right)+r_{1B}B^{*}\left(t\right)$$

(9) $$\mathrm{dC}o\left(t\right)/dt=-k_{\mathrm{AB}}(A\left(t\right)+A^{*}\left(t\right))\mathrm{Co}\left(t\right)$$

This can be reduced to pseudo‐first‐order kinetics by introducing a time‐dependent rate constant:

(10) $${\mathrm{dA}}^{*}\left(t\right)/dt=-k_{\mathrm{AB}}^{'}\left(t\right)A^{*}\left(t\right)-r_{1A}A^{*}\left(t\right)$$

etc.

where $\;k_{\mathrm{AB}}^{'}\left(t\right)=k_{\mathrm{AB}}\mathrm{Co}\left(t\right)$.

**Figure 1:**
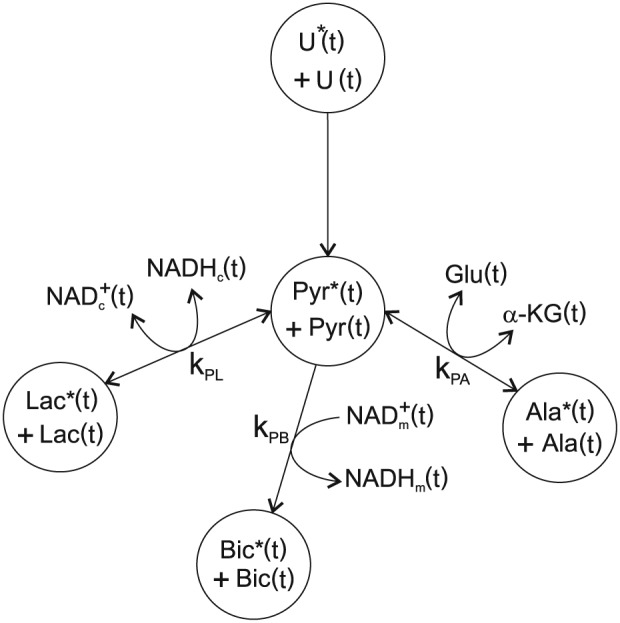
Schematic of the metabolic model describing the conversion of hyperpolarized and unpolarized pyruvate (P* + P) to lactate (L* and L), alanine (A* and A) and bicarbonate (B* and B). The rate constants k_PL_, k_PA_ are assumed the same for forward and reverse reactions whereas the pools of cofactors NAD^+^_c_(t), NADH_c_(t), Glu(t), α‐KG(t), NAD^+^_m_(t) and NADH_m_(t) are freely varying in the model.

Employing the above formalism, a system of differential equations can be written for reactions of hyperpolarized pyruvate in the heart, shown schematically in Figure 1, including the exchange reaction of pyruvate–lactate, mediated by LDH, the exchange reaction of pyruvate–alanine, mediated by ALT, and the one way flux of pyruvate–CO_2_, mediated by PDH and subsequent carbonic anhydrase (and pH)‐mediated interconversion of CO_2_ to bicarbonate, which we model as a single flux from pyruvate–bicarbonate, as follows.

(11) $${\mathrm{dP}}^{*}\left(t\right)/dt=-k{'}_{\mathrm{PL}}\left(t\right)P^{*}\left(t\right)+k{'}_{\mathrm{LP}}\left(t\right)L^{*}\left(t\right)-k{'}_{\mathrm{PA}}\left(t\right)P^{*}\left(t\right)+k{'}_{\mathrm{AP}}\left(t\right)A^{*}\left(t\right)-k{'}_{\mathrm{PB}}\left(t\right)P^{*}\left(t\right)-r_{1P}P^{*}\left(t\right)+U^{*}\left(t\right)$$

(12) $$dL^{*}\left(t\right)/dt=k{'}_{\mathrm{PL}}\left(t\right)P^{*}\left(t\right)-k{'}_{\mathrm{LP}}\left(t\right)L^{*}\left(t\right)-r_{1L}L^{*}\left(t\right)$$

(13) $${\mathrm{dA}}^{*}\left(t\right)/dt=k{'}_{\mathrm{PA}}\left(t\right)P^{*}\left(t\right)-k{'}_{\mathrm{AP}}\left(t\right)A^{*}\left(t\right)-r_{1A}A^{*}\left(t\right)$$

(14) $${\mathrm{dB}}^{*}\left(t\right)/dt=k{'}_{\mathrm{PB}}\left(t\right)P^{*}\left(t\right)-r_{1B}B^{*}\left(t\right)$$

(15) $$dP\left(t\right)/dt\;=-k{'}_{\mathrm{PL}}\left(t\right)P\left(t\right)+k{'}_{\mathrm{LP}}\left(t\right)L\left(t\right)-k{'}_{\mathrm{PA}}\left(t\right)P\left(t\right)+k{'}_{\mathrm{AP}}\left(t\right)A\left(t\right)-k{'}_{\mathrm{PB}}\left(t\right)P\left(t\right)+r_{1P}P^{*}\left(t\right)+U\left(t\right)$$

(16) $$\mathrm{dL}\left(t\right)/dt\;=k{'}_{\mathrm{PL}}\left(t\right)P\left(t\right)-k{'}_{\mathrm{LP}}\left(t\right)L\left(t\right)+r_{1L}L^{*}\left(t\right)$$

(17) $$\mathrm{dA}\left(t\right)/dt=k{'}_{\mathrm{PA}}\left(t\right)P\left(t\right)-k{'}_{\mathrm{AP}}\left(t\right)A\left(t\right)+r_{1A}A^{*}\left(t\right)$$

(18) $$\mathrm{dB}\left(t\right)/dt=k{'}_{\mathrm{PB}}\left(t\right)P\left(t\right)+r_{1B}B^{*}\left(t\right)$$

(19) $${\text{dNAD}}_{c}\left(t\right)/dt=k{'}_{\mathrm{PL}}\left(t\right)P^{*}\left(t\right)+k{'}_{\mathrm{PL}}\left(t\right)P\left(t\right)-k{'}_{\mathrm{LP}}\left(t\right)L^{*}\left(t\right)-k{'}_{\mathrm{LP}}\left(t\right)L\left(t\right)$$

(20) $${\text{dNADH}}_{c}\left(t\right)/dt=-k{'}_{\mathrm{PL}}\left(t\right)P^{*}\left(t\right)-k{'}_{\mathrm{PL}}\left(t\right)P\left(t\right)+k{'}_{\mathrm{LP}}\left(t\right)L^{*}\left(t\right)+k{'}_{\mathrm{LP}}\left(t\right)L\left(t\right)$$

(21) $$\text{dGlu}\left(t\right)/dt=-k{'}_{\mathrm{PA}}\left(t\right)P^{*}\left(t\right)-k{'}_{\mathrm{PA}}\left(t\right)P\left(t\right)+k{'}_{\mathrm{AP}}\left(t\right)A^{*}\left(t\right)+k{'}_{\mathrm{AP}}\left(t\right)A\left(t\right)$$

(22) $$\text{dαKG}\left(t\right)/dt=k{'}_{\mathrm{PA}}\left(t\right)P^{*}\left(t\right)+k{'}_{\mathrm{PA}}\left(t\right)P\left(t\right)-k{'}_{\mathrm{AP}}\left(t\right)A^{*}\left(t\right)-k{'}_{\mathrm{AP}}\left(t\right)A\left(t\right)$$

(23) $${\text{dNAD}}_{m}\left(t\right)/dt=-k{'}_{\mathrm{PB}}\left(t\right)P^{*}\left(t\right)-k{'}_{\mathrm{PB}}\left(t\right)P\left(t\right)$$

(24) $${\text{dNADH}}_{m}\left(t\right)/dt=k{'}_{\mathrm{PB}}\left(t\right)P^{*}\left(t\right)+k{'}_{\mathrm{PB}}\left(t\right)P\left(t\right)$$

Hyperpolarized substrate pools are given by an * and all other substrates and cofactors are non‐hyperpolarized. The pyruvate pool is given by P*(*t*) + P(*t*) with an effective longitudinal relaxation rate *r*_1P_, alanine is given by A*(*t*) + A(*t*) with an effective longitudinal relaxation rate *r*_1A_, lactate is given by L*(*t*) + L(*t*) with an effective longitudinal relaxation rate *r*_1L_ and bicarbonate is given by B*(*t*) + B(*t*) with an effective longitudinal relaxation rate *r*_1B_. In the model the pseudo rate constants for forward and reverse reactions were defined as a product of a second‐order rate constant and a time‐dependent concentration term, *k*_PL_′(*t*) = *k*_PL_NADH_c_(*t*), *k*_LP_′(*t*) = *k*_PL_NAD_c_(*t*), *k*_PA_′(*t*) = *k*_PA_Glu(*t*), *k*_AP_′(*t*) = *k*_PA_αKG(*t*), *k*_PB_′(*t*) = *k*_PB_NADH_m_(*t*), allowing the pseudo time‐dependent rate constants to be different in forward and reverse reactions. Cytosolic NAD^+^ and NADH are given by NAD_c_(*t*) and NADH_c_(*t*) respectively, glutamate is given by Glu(*t*), α‐ketoglutarate is given by α‐KG(*t*) and mitochondrial NAD^+^ and NADH are given by NAD_m_(*t*) and NADH_m_(*t*), respectively. It should be noted however that the concentrations defined using this phenomenological approach do not correspond to free cofactor concentrations, since we have not included a full description of the enzyme–cofactor binding kinetics 15.

Fitting to a first‐order model was carried out by removing the cofactor terms from Equations [(11)]–[(27)] and substituting the pseudo rate constants *k*_PL_′(*t*) etc. for time‐independent first‐order rate constants *k*_PL_, *k*_LP_ etc.

The injection bolus was modeled as a gamma variate function U*(*t*) that decays to a non‐hyperpolarized input function *U*(*t*) given by.

(25) $$U^{*}\left(t\right)=k_{\mathrm{in}}{\left(t-t_{\text{arrival}}\right)}^{\delta }e^{-\beta \left(t-t_{\text{arrival}}\right)}$$

(26) $$U\left(t\right)=k_{\mathrm{in}}\int_{0}^{t}\beta {\left(t-t_{\text{arrival}}\right)}^{\delta }e^{-\beta \left(t-t_{\text{arrival}}\right)}\mathrm{dt}$$

(27) $$dU\left(t\right)/dt=\beta U^{*}\left(t\right)$$

where *t*_arrival_ is the time point at which the hyperpolarized signal of pyruvate is first detected, *δ* and *β* describe the rise and fall of the input function and *k*_in_ is the amplitude.

### Langendorff heart perfusion

Hearts from male Wistar rats (220–250 g, *n* = 5/group) were excised and retrograde perfused in Langendorff mode with a modified Krebs buffer 25 containing 118 mM NaCl, 25 mM NaHCO_3_, 1.2 mM MgSO_4_, 5.9 mM KCl, 0.6 mM Na_2_EDTA, 11.1 mM glucose and 2.5 mM CaCl_2_, pH 7.4, equilibrated with a 95% O_2_, 5% CO_2_ gas mixture at 37 °C. Coronary flow at the heart was maintained at a constant 14 ml/min to achieve a physiological perfusion pressure of approximately 76 mm Hg, monitored by an in‐line pressure transducer. The perfused heart was placed in a glass tube in the isocentre of a ^23^Na–^13^C saddle coil (15 mm) and inserted into the vertical bore of a Bruker 9.4 T AVANCE III spectrometer. The temperature within the scanner was maintained at 37 °C by warming the imaging gradients and calibrating the temperature at the position of the heart using an ethylene glycol capillary. After a stabilization period of 10 min, hearts were either maintained on the control buffer described above (the glucose only group), or switched to a second buffer supplemented with 2.5 mM pyruvate for the remainder of the experiment (the pyruvate supplemented group).

### Hyperpolarized ^13^C NMR spectroscopy

Pyruvic acid containing 15 mM trityl radical and 1 mM gadolinium (Dotarem) was hyperpolarized in a HyperSense® (Oxford Instruments) DNP at 3.35 T and 1.4 K for about 1 h. The protocol typically resulted in a 20 000‐fold enhancement (corresponding to a polarization *P* = 16%) in the ^13^C MR signal from [1‐^13^C] pyruvate. Dissolution of the hyperpolarized pyruvate was carried out in 4 ml of glucose free Krebs buffer containing NaOH and EDTA to achieve a final solution of 50 mM hyperpolarized pyruvate at pH = 7. The hyperpolarized solution was injected into the arterial perfusion line via a side‐arm below the bore of the NMR magnet at a constant flow (1 ml/min) using an automated injector. The final concentration of hyperpolarized pyruvate arriving at the heart was 3.3 mM. Serial ^13^C‐MRS spectra were acquired with a time interval of 2 s using a small flip angle pulse (*θ* = 10°). Spectra were baseline corrected and metabolite peaks of lactate, alanine, bicarbonate and pyruvate were integrated.

### Data analysis

All hyperpolarized time series were first normalized to the maximum pyruvate peak area obtained in each experiment. The hyperpolarized signals P*_i_, L*_i_, A*_i_ and B*_i_ measured at times *t*_1_, *t*_2_, …, *t*_*N*_ were then fit simultaneously to the differential equations [(10)]–[(26)], using the ordinary differential equation solver in MATLAB (MathWorks**®)** assuming zero signal at *t* < *t*_arrival_ for all hyperpolarized and non‐polarized substrates. All variables *k*_PL_, *k*_PA_, *k*_PB_, *r*_1L_, *r*_1A_, *r*_1B_, *r*_1P_, *k*_in_, *t*_arrival_, δ and *β*, as well as initial cofactor concentrations NAD_c_(0), NADH_c_(0), Glu(0), α‐KG(0), NAD_m_(0) and NADH_m_(0), were allowed to vary freely within given boundary conditions. The noise variance was different for the four measured signals, so a cost function derived from a maximum likelihood analysis was used for data fitting. Gaussian noise was assumed with separate variances on each signal. The log‐likelihood function was then maximized with respect to each variance, leading to the following cost function (to be minimized):

(28) $$\begin{array}{l} \text{cost}=\text{log}\left(\sum_{i=1}^{N}{\left(P^{*}\left(t_{i}\right)-{P^{*}}_{i}\right)}^{2}\right)+\text{log}\left(\sum_{i=1}^{N}{\left(L^{*}\left(t_{i}\right)-{L^{*}}_{i}\right)}^{2}\right)+\text{log}\left(\sum_{i=1}^{N}{\left(A^{*}\left(t_{i}\right)-{A^{*}}_{i}\right)}^{2}\right)\\ +\text{log}\left(\sum_{i=1}^{N}{\left(B^{*}\left(t_{i}\right)-{B^{*}}_{i}\right)}^{2}\right) \end{array}$$

where P*(*t*_*i*_) etc. are the signal values predicted from the model.

Where appropriate, statistics were performed using Student's *t* test, and *P* < 0.05 was taken to be significant.

### NADH surface fluorescence

NADH surface fluorescence experiments were performed in two further groups of rat hearts (*n* = 4 per group) perfused under identical conditions to the hyperpolarized experiments, varying only in that the infused pyruvate solution lacked the trityl free radical. End‐diastolic pressure was set at 4–8 mm Hg using a water filled polyethylene balloon inserted into the left ventricular cavity. The hearts were continuously submerged in 37 °C perfusate within a custom built imaging chamber. NADH surface fluorescence of the left ventricle was recorded with a video fluorometer, as previously reported^26^, ^27^. Briefly, the fluorometer consisted of a 100 W mercury lamp, and a camera unit housing a dichroic mirror for a digital video recorder. The fluorometer was positioned in front of the crystal window of the imaging chamber that contained the submerged heart. The length of the experimental protocol was 600 s: following 60 s of baseline perfusion, pyruvate was infused for 180 s, followed by a 180 s recovery period and then 180 s of total no‐flow ischemia. NADH images were recorded in the periods 0–30 s (baseline), 60–240 s (during pyruvate infusion), 390–420 s (recovery of baseline), and 590–600 s (end ischemia). The fluorescence images were analyzed offline with MATLAB software, where the largest area of the left ventricle visible throughout the whole experiment was chosen and the averaged grey level determined and corrected for fluctuations in lamp intensities by normalization with the fluorescence intensity of a piece of uranyl positioned next to the submerged heart and decay corrected to account for photo‐bleaching effects. NADH fluorescence was normalized to the maximal NADH fluorescence obtained at the end of the ischemic period in each experiment.

## Results

**Figure 2:**
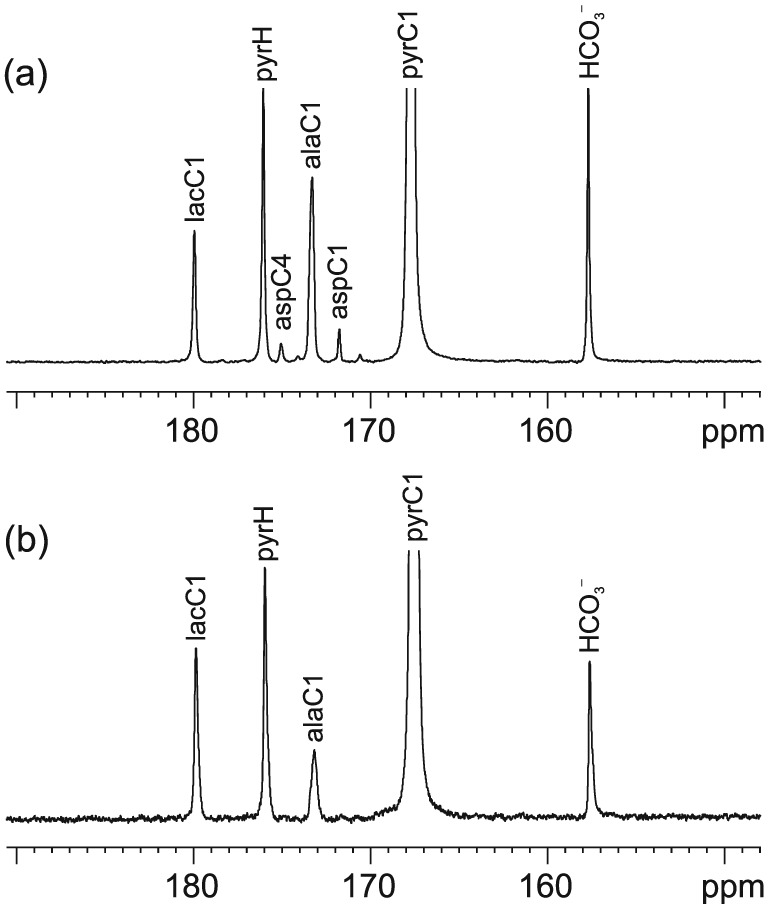
A representative summed hyperpolarized ^13^C spectra from isolated rat hearts perfused with Krebs buffer containing (a) 10 mM glucose and (b) 10 mM glucose supplemented with 2.5 mM pyruvate. The spectra display peaks from the injected pyruvate, pyruvate hydrate, lactate, alanine, bicarbonate and CO_2_. In addition the hearts in (a) also displayed peaks from C1 and C4 aspartate which were absent in (b).

Figure 2 shows representative hyperpolarized ^13^C spectra summed across the entire time series for (a) glucose only hearts and (b) glucose + pyruvate supplemented hearts. All spectra displayed metabolite peaks from the parent pyruvate, pyruvate hydrate, lactate, alanine, bicarbonate and CO_2_. The ^13^C spectra from hearts perfused with glucose only also exhibited two further peaks at about 175 parts per million (ppm) and at about 172 ppm, which did not appear in spectra from hearts supplemented with pyruvate. These peaks have been previously attributed to the C1 and C4 carbons of aspartate 28, 29.

**Figure 3:**
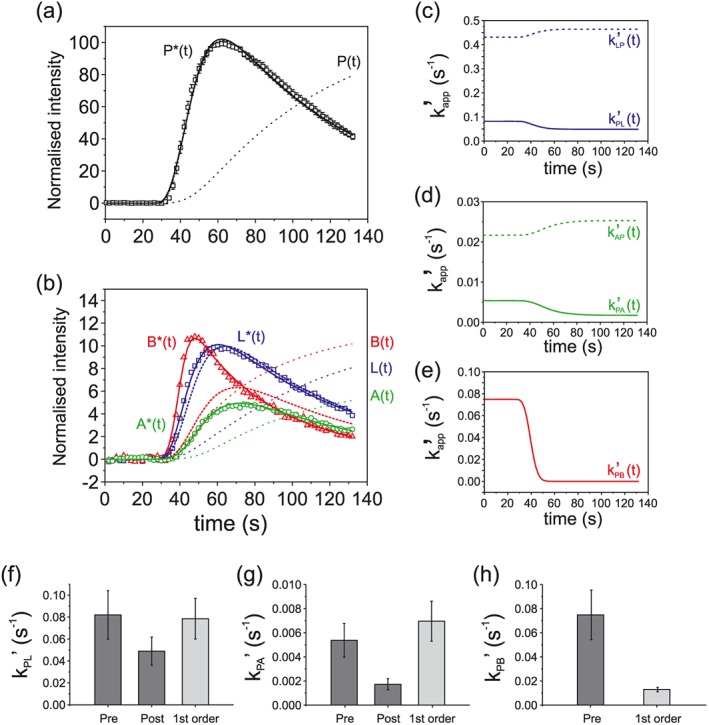
(a), (b) Representative fits of the glucose only perfused hearts showing (a) the hyperpolarized pyruvate curves P* (thick solid black line) and the resulting non‐polarized pyruvate pool P (dashed line), (b) the hyperpolarized lactate L*(*t*), alanine A*(*t*) and bicarbonate B*(*t*) (blue, green and red thick solid lines, respectively) with the resulting non‐polarized pools L(*t*), A(*t*) and B(*t*) (red, green and blue dashed lines, respectively). Overlaid first‐order fits are also shown (thick dashed lines). (c)–(e) The pseudo rate constants *k*_PL_′(*t*) (solid line) and *k*_LP_′(*t*) (dashed line) are shown in (c), *k*_PA_′(*t*) (solid line) and *k*_AP_′(*t*) (dashed line) in (d) and *k*_PB_′(*t*) (solid line) in (e). (f)–(h) Mean rate constants *k*_PL_′(*t*), *k*_PA_′(*t*) and k_PB_′(*t*), pre hyperpolarized pyruvate injection and post pyruvate injection derived from the second‐order and first‐order fitting (*n* = 5, ±SEM).

**Figure 4:**
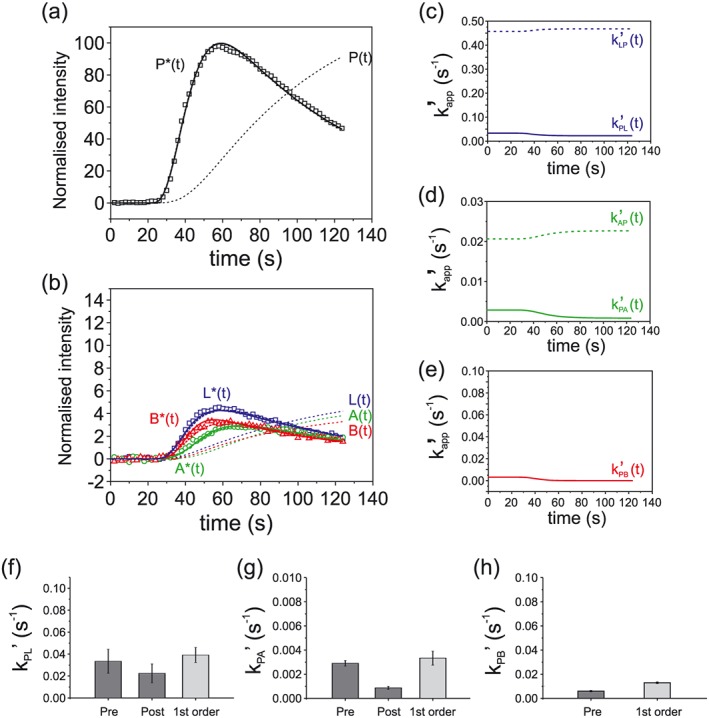
(a), (b) Representative fits of the pyruvate supplemented perfused hearts showing (a) the hyperpolarized pyruvate curves P* (thick solid black line) and the resulting non‐polarized pyruvate pool P (dashed line), (b) the hyperpolarized lactate L*(*t*), alanine A*(*t*) and bicarbonate B*(*t*) (blue, green and red thick solid lines, respectively) with the resulting non‐polarized pools L(*t*), A(*t*) and B(*t*) (red, green and blue dashed lines, respectively). Overlaid first‐order fits are also shown (thick dashed lines). (c)–(e) The pseudo rate constants *k*_PL_′(*t*) (solid line) and *k*_LP_′(*t*) (dashed line) are shown in (c), *k*_PA_′(*t*) (solid line) and *k*_AP_′(t) (dashed line) in (d) and *k*_PB_′(*t*) (solid line) in (e). (f)–(h) Mean pseudo rate constants *k*_PL_′(*t*), *k*_PA_′(*t*) and *k*_PB_′(*t*), pre hyperpolarized pyruvate injection and post pyruvate injection derived from the second‐order and from first‐order fitting (*n* = 5, ±SEM).

Fits from the glucose only KHB perfused hearts and the glucose + pyruvate supplemented hearts are shown in Figures 3 and 4, respectively, presented on the same vertical axis. Second‐order fits are shown in Figures 3, 4(a), (b), for the hyperpolarized pyruvate P* (thick black solid line), lactate L*(*t*) (thick blue solid line), alanine A*(*t*) (thick green solid line) and bicarbonate B*(*t*) (thick red solid line). Also shown in Figures 3, 4 are the resulting non‐polarized pools P(*t*), L(*t*), A(*t*) and B(*t*) (black, blue, green and red thin dashed lines, respectively) and first‐order fits for P*(*t*), L*(*t*), A*(*t*) and B*(*t*) (black, blue, green and red thick dashed lines, respectively). The time‐dependent pseudo rate constants derived from the second‐order modeling are shown in Figures 3, 4(c)–(e), where *k*_PL_′(*t*) and *k*_LP_′(*t*) are the forward and reverse reactions of pyruvate to lactate (solid and dashed blue lines, respectively), *k*_PA_′(*t*) and *k*_AP_′(*t*) are the forward and reverse reactions of pyruvate to alanine (solid and dashed green lines, respectively), and *k*_PB_′(*t*) is the forward reaction of pyruvate to bicarbonate (solid red line). In both groups of hearts the second‐order model yielded a time‐dependent decrease in the pseudo rate constants for the reaction of pyruvate to bicarbonate *k*_PB_′(*t*) as well as for the forward reactions of pyruvate to lactate *k*_PL_′(*t*) and pyruvate to alanine *k*_PA_′(*t*), whilst the model exhibited a time‐dependent increase in the pseudo rate constants for the reverse reactions of lactate to pyruvate *k*_LP_′(*t*) and alanine to pyruvate *k*_AP_′(*t*). Figures 3, 4(f)–(h) show the rate constants (mean ± standard error of the mean (SEM), *n* = 5 per group) pre and post pyruvate infusion derived from the second‐order model and the corresponding rate constants derived from the first‐order model (Table 1). The pseudo rate constant for pyruvate to bicarbonate conversion was significantly lower in the pyruvate supplemented hearts, *k*_PB_′(*t*) = 0.0059 ± 0.0004, than in those supplied with glucose only, *k*_PB_′(*t*) = 0.075 ± 0.021 (*P = 0.028, Student's *t* test). The first‐order rate constants *k*_PB_ deviated significantly from those estimated from the second‐order model, being underestimated by the first‐order model in the glucose only hearts and overestimated in the pyruvate supplemented hearts, reflecting the poor fits using the first‐order model. First‐order rate constants *k*_PL_ and *k*_PA_ were similar in magnitude to the pre‐injection rate constants *k*_PL_′t) and *k*_PA_′(*t*).

**Table 1:** Kinetic parameters derived from second‐order modeling employing Equations [(10)]–[(26)] and the full model described in Figure 1 as well as corresponding kinetic parameters derived from a first‐order model

	Glucose only	*R* ^2^ (*n* = 5)	Pyruvate supplemented	*R* ^2^ (*n* = 5)
*k*_PL_′(Pre)	0.082 ± 0.022	0.957 ± 0.033	0.034 ± 0.011	0.968 ± 0.011
*k*_PL_′(Post)	0.049 ± 0.013		0.023 ± 0.009	
*k*_PL_ (first order)	0.083 ± 0.020	0.950 ± 0.030	0.041 ± 0.005	0.960 ± 0.011
*k*_PA_′(Pre)	0.005 ± 0.001	0.971 ± 0.016	0.0029 ± 0.0002	0.951 ± 0.018
*k*_PA_′(Post)	0.0017 ± 0.0005		0.0009 ± 0.0001	
*k*_PA_ (first order)	0.007 ± 0.0017	0.971 ± 0.026	0.003 ± 0.0006	0.949 ± 0.039
*k*_PB_′(Pre)	0.075 ± 0.021	0.978 ± 0.008	0.0059 ± 0.0004	0.931 ± 0.022
*k*_PB_′(Post)	−7.9 × 10^−12^ ± 8.7 × 10^−12^		6.7 × 10^−9^ ± 4.0 × 10^−9^	
*k*_PB_ (first order)	0.016 ± 0.0003	0.539 ± 0.283	0.013 ± 0.0007	0.925 ± 0.048

The values of *T*_1_ for each of the metabolites derived from the fitting were not well determined using the first‐order modeling due to the relaxation rates being strongly correlated with the rate constants for the reverse reactions, as previously demonstrated in the literature 30. However, for the second‐order model the relaxation times were better characterized. From our study for the glucose only perfused hearts, the relaxation times returned from the fits were *T*_1L_ = 44.3 ± 3.7 s, *T*_1A_ = 60.0 ± 7.6 s, *T*_1B_ = 50.5 ± 6.1 s, *T*_1P_ = 76.7 ± 4.4 s (±SEM, *n* = 5). For the pyruvate supplemented hearts, the relaxation rates returned from the fits were *T*_1L_ = 40.2 ± 4.6 s, *T*_1A_ = 68.0 ± 8.6 s, *T*_1B_ = 73.4 ± 3.1 s, *T*_1P_ = 76.0 ± 5.0 s (±SEM, *n* = 5). Since our hearts are perfused with a crystalloid buffer these relaxation times will be longer than *in vivo* or in a blood perfused heart, but nonetheless are close to values previously reported *in vitro* for each metabolite of *T*_1L_ = 45 s (at 3 T), *T*_1A_ = 42 s (at 3 T), *T*_1B_ = 50 s (at 3 T), *T*_1P_ = 67 s (at 3 T) 16.

**Figure 5:**
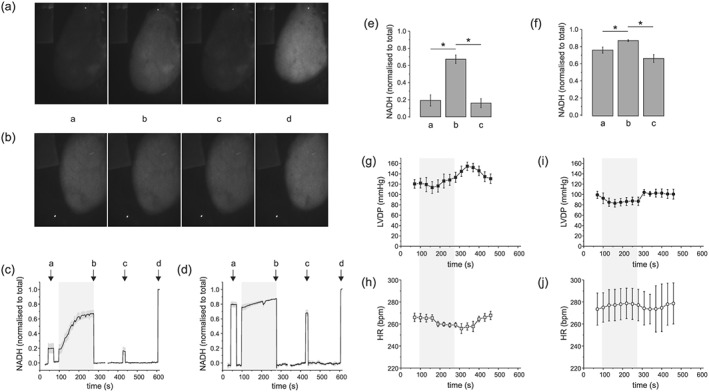
(a), (b) Representative NADH surface fluorescence images from (a) glucose only perfused heart and (b) glucose + pyruvate supplemented heart at time points a corresponding to the baseline pre pyruvate infusion, b at the end of the pyruvate infusion, c post stopping the infusion and d during global stopped flow ischemia. (c), (d) NADH surface fluorescence intensity from the left ventricle normalized to the total during ischemia in glucose only perfused hearts and in pyruvate supplemented perfused hearts, respectively. (e), (f) Mean NADH fluorescence intensity normalized to the total NADH fluorescence (measured during global ischemia) at the time points a before pyruvate infusion, b at the end of the pyruvate infusion and c post stopping the pyruvate infusion in the two groups, respectively (*P < 0.05, *n* = 4). (g), (h) and (i), (j) Functional measurements of LVDP and HR in the two groups, respectively.

Figure 5 shows the NADH surface fluorescence measurements under the same conditions as the hyperpolarized experiments, i.e. perfusion with buffer containing glucose only (a) and (c), or glucose supplemented with 2.5 mM pyruvate (b) and (d), with an additional 3 min infusion of 50 mM pyruvate (denoted by the shaded area). Representative whole heart NADH surface fluorescence images are shown at time points a before pyruvate infusion, b at the end of the pyruvate infusion, c post stopping the pyruvate infusion and d during global ischemia in the two groups. Figure 5(e), (f) shows the mean NADH fluorescence intensity normalized to the total NADH fluorescence (measured during global ischemia) at the time points a before pyruvate infusion, b at the end of the pyruvate infusion and c post stopping the pyruvate infusion in the two groups, respectively. In both groups of hearts there was a significant increase of NADH signal (*P < 0.05) at the end of the pyruvate infusion, time point b. In the glucose only perfused hearts normalized NADH was 0.19 ± 0.06 at baseline, increasing to 0.67 ± 0.05 at the end of the pyruvate infusion, returning to 0.16 ± 0.05 post stopping the infusion. In the pyruvate supplemented hearts normalized NADH was 0.76 ± 0.04 at baseline, increasing to 0.87 ± 0.01 at the end of the pyruvate infusion, returning to 0.66 ± 0.04 post stopping the infusion. In both groups of hearts the NADH signal post stopping the infusion, time point c, recovered to and was not significantly different from the baseline NADH signal measured before pyruvate infusion. Baseline NADH fluorescence, at time point a, was significantly greater in the pyruvate supplemented hearts than in those perfused with glucose alone. Left ventricular developed pressure (LVDP) and heart rate (HR) are shown in Figure 5(g), (h) and (i), (j), respectively, shown on the same timescale as the NADH surface fluorescence measurements. Cardiac function was not significantly perturbed during pyruvate infusion.

## Discussion

We have developed a modeling approach to assess possible non‐linear kinetics of hyperpolarized pyruvate in the crystalloid perfused rat heart. A second‐order model yielded good agreement with the hyperpolarized ^13^C experimental data in two groups of hearts perfused with either glucose only buffer or glucose buffer supplemented with pyruvate. We were simultaneously able to fit pyruvate, lactate, alanine and bicarbonate dynamics, suggesting non‐linear kinetics during the infusion of hyperpolarized pyruvate. In the case of the pyruvate to bicarbonate kinetics the data was poorly fit by a first‐order model in the glucose only perfused hearts (*R* ^2^ = 0.539 ± 0.283), which was significantly improved in the second‐order model (*R* ^2^ = 0.978 ± 0.008). Second‐order fitting also led to an improvement in the bicarbonate fitting of the pyruvate supplemented hearts (*R* ^2^ = 0.931 ± 0.022) compared with first‐order fitting (*R* ^2^ = 0.925 ± 0.048) but was not significant. Pyruvate to lactate fits were improved in the glucose only perfused hearts (*R* ^2^ = 0.957 ± 0.033) with respect to the first‐order model (*R* ^2^ = 0.950 ± 0.030) and also improved by second‐order modeling in the pyruvate supplemented hearts (*R* ^2^ = 0.968 ± 0.011) with respect to first‐order modeling (*R* ^2^ = 0.960 ± 0.011), although in neither case was this significant. Pyruvate to alanine kinetics were not improved by second‐order fitting and were indistinguishable from the first‐order fits in both groups of hearts.

In both groups of hearts the second‐order model suggested a time‐dependent decrease in flux through the PDH complex during the pyruvate infusion, which could reflect a perturbation due to the change in substrate concentration during the infusion and subsequent shift of steady‐state equilibrium kinetics, but other factors may also play a role, including change in enzyme activity due to substrate inhibition or a change in activity due to exhaustion of cofactor availability, for example NAD^+^. It has previously been shown that infusion of pyruvate in the perfused heart leads to an increase in NADH 19, 20, which was attributed to increased flux of pyruvate into the TCA cycle and subsequent increased NADH production. However, in our experiments the flux of pyruvate to bicarbonate was significantly lower in the pyruvate supplemented hearts than in those supplied with glucose only. Since the PDH reaction is uni‐directional, we postulated that pyruvate infusion leads to a depletion of mitochondrial NAD^+^ (conversion to NADH) and that the flux is therefore dependent on the availability of this cofactor, leading to a reduction in the rate.

To investigate this further, we measured NADH surface autofluorescence in parallel groups of hearts perfused under the same conditions as for the hyperpolarized experiments. Surface autofluorescence measurements of NADH have previously been shown to be dominated by mitochondrial NADH 31. Our experiments showed a significant time‐dependent increase in NADH during the pyruvate infusion in both groups of hearts and a significantly elevated baseline NADH in pyruvate supplemented hearts, consistent with previous studies 19, 20. Assuming the total pool size of mitochondrial NAD^+^ + NADH to be constant within the timescale of the infusion, then the time‐dependent increase in NADH infers a concomitant time‐dependent decrease of NAD^+^ during pyruvate infusion as well as a reduced baseline NAD^+^ in the pyruvate supplemented hearts, supporting our hypothesis that the kinetics of hyperpolarized [1‐^13^C] pyruvate through PDH is non‐linear and subject to the decreased availability of mitochondrial NAD^+^. Cardiac function reflected by HR and LVDP was not significantly perturbed during the infusion and NADH surface fluorescence returned to baseline values after stopping the infusion in both groups of hearts, suggesting this to be a transient effect.

The second‐order model suggested non‐linear kinetics and a time‐dependent decrease in the derived pseudo rate constants for the forward reaction of both pyruvate to lactate and pyruvate to alanine. Conversely, the model suggested a time‐dependent increase of the pseudo rate constants for the reverse reactions. However there was no significant difference between the goodnesses of fit (*R* ^2^) for the two models, and from the fitting alone it is not possible to distinguish between first‐ and second‐order models from the current data. This suggests that cofactor concentrations may not limiting in either case due to the reversible nature of the enzyme reactions catalyzed by LDH and ALT and subsequent regeneration of these cofactors in either forward or reverse reactions.

The fits returned values of the reverse rate constants that were greater than the forward rate constants for both *k*′_LP_ and *k*′_AP_. To investigate this further we also performed fits to one‐way kinetics, i.e. no backreaction, and in the case of both lactate and alanine a one‐way model also gave satisfactory fits (data not shown). The backreaction kinetics have previously been shown to be strongly correlated with the relaxation rates, introducing significant errors in estimation of these parameters. The greater magnitude of these rate constants in the current data likely reflects the modeling rather than underlying physiology. Measurement of the reverse exchange rate constants has recently been addressed using magnetization exchange experiments 32; experiments employing hyperpolarized lactate 33 or alanine 34 could also be performed.

The aspartate peaks evident in glucose only perfused hearts were absent from pyruvate supplemented hearts. The appearance of the C4 aspartate peak has been previously attributed to scrambling of the C1 and C4 labels due interconversion with fumarate in the TCA cycle (due to its symmetric nature). However the absence of these peaks in the pyruvate supplemented hearts is worth noting, since the malate–aspartate shuttle is responsible for translocating electrons from cytosolic NADH across the inner mitochondrial membrane. Since this shuttle is dependent upon NAD^+^/NADH ratio, the elevated baseline NADH that we observe in these hearts using surface fluorescence would lead to a reduced requirement to translocate electrons from glycolytically derived NADH from the cytosol into the mitochondria, and therefore potentially reduced activity of the malate–aspartate shuttle in the pyruvate supplemented hearts.

To date much work has been carried out to explore the utility of hyperpolarized substrates as a measure of metabolic fluxes or exchange rates of a number of enzymes and transporters in biological systems including the heart, cancer, the liver and the brain, in *ex. vivo* perfused models such as that presented here as well as *in vitro* cell systems or *in vivo* disease models. Successes have been attributed to the ability to measure changes in kinetics either in response to disease progression or employing the method as a potential biomarker of treatment response 35. Results have typically been interpreted as a measure of fluxes or exchange using first order models. Our kinetic data obtained from the perfused heart suggests that the assumption that these measurements display first‐order kinetics may not always hold, particularly for PDH in a highly metabolically active and flexible organ such as the perfused heart. In our experiments we infused a solution of 50 mM hyperpolarized pyruvate, which resulted in 3.3 mM hyperpolarized pyruvate arriving at the myocardium after dilution in two groups of hearts, perfused with glucose only buffer in the absence of pyruvate or supplemented with 2.5 mM pyruvate. These concentrations greatly exceed the endogenous concentration of pyruvate in the blood (typically about 0.1 mM 36) and therefore resulted in a metabolic perturbation in our experiments due to a change in substrate supply during infusion, accounting for the non‐linear response. Employing second‐order models may yield novel insights, particularly with respect to the importance of enzyme cofactor pools in the measured kinetics.

We conclude that infusion of supra‐physiological concentrations of pyruvate into the glucose only and glucose + pyruvate supplemented perfused hearts leads to a transient perturbation of the steady‐state kinetics of PDH that leads to non‐linear dynamics and non‐first‐order kinetics, likely related to exhaustion of NAD^+^ as a cofactor. For lactate and alanine kinetics we were not able to distinguish between first‐ and second‐order models and it was not conclusive from the current data whether the infusion also led to a perturbation of the enzyme equilibria of these reactions. These are potentially important considerations when utilizing and interpreting NMR data obtained following infusion of non‐physiological concentrations of hyperpolarized substrates.
